# Editorial: Myopathology of inherited myopathies

**DOI:** 10.3389/fneur.2022.1004562

**Published:** 2022-08-17

**Authors:** Chiara Fiorillo, Edoardo Malfatti, Giovanni Meola

**Affiliations:** ^1^Neurologia Pediatrica e Malattie Muscolari, IRCCS Istituto Giannina Gaslini, Dinogmi University of Genoa, Genoa, Italy; ^2^Université Paris Est, U955 INSERM, EnvA, EFS, IMRB, Créteil, France; ^3^APHP, Centre de Référence de Pathologie Neuromusculaire Nord-Est-Ile-de-France, Henri Mondor Hospital, Créteil, France; ^4^Department of Biomedical Sciences, University of Milan, Milan, Italy; ^5^Department of Neurorehabilitation Sciences, Casa di Cura del Policlinico, Milan, Italy

**Keywords:** muscle biopsy, NGS, congenital myopathies, floppy infant, *TNPO3*, RYR1, *MATR3*

The inherited myopathies are a large group of neuromuscular disorders belonging to different groups classified on the basis of histopathologic lesions found in muscle biopsy (e.g., congenital structural myopathies, myofibrillar myopathies), the defective enzyme/protein (e.g., muscle glycogenoses; mitochondrial disorders), or the distribution of the muscular weakness (e.g., limb girdle muscular dystrophy).

Given the expanding number of genes involved, the NGS approach is considered the most effective diagnostic tool. However, these techniques are not available everywhere and carry a burdensome bioinformatic analysis that hinders or delays the achievement of a clear diagnosis. Moreover, NGS studies often identify multiple genetic variants in different genes that need a functional validation of pathogenicity.

In this scenario, the muscle biopsy renews its importance as being able to provide diagnostic indications in a short amount of time, and allowing the performance of different techniques spanning from histochemistry, immunostochemistry, electron microscopy, and biochemical studies. The combined use of these techniques provides a complete view of the muscle lesions and supports the interpretation of variants of uncertain significance (VUS).

Our call for contributions started in early 2021 and resulted in 8 peer-reviewed publications covering a wide range of topics in the field of inherited myopathies, with a particular focus on translational myopathology.

Costa et al. reported a comprehensive review of the pathogenetic aspect of the dominant LGMD caused by mutation in the *TNPO3* gene (LGMD D2). *TNPO3* encodes the transportin-3 (TPNO3), a protein belonging to the importin beta-superfamily. TNPO3 normally mediates the nuclear import of Ser/Arg-rich proteins involved in mRNA metabolism and splicing. To date, the role of TNPO3 in skeletal muscle, as well as the disease pathomechanisms leading to clinical manifestation, need to be elucidated. The limited number of affected patients, the variable clinical features, and the differences in the pattern of histopathological lesions hinder the identification of the exact pathomechanism. The authors of this comprehensive review propose three different mechanisms: 1. The mutated *TNPO3* is no longer able to bind its cargoes or is not able to transport them to the nucleus correctly, thus altering the physiologic functions. In this scenario, LGMD2 can be considered among the nuclear envelopathies such as the laminopathies. 2. The altered TPNO3 leads to altered protein turnover with myofibrillar disarray and surcharge, such as in myofibrillar myopathies, and autophagy might have a role in myofiber atrophy and is associated with the progression of the disorder. 3. Pathomechanism is a dominant-negative effect of mutated *TPNO3* on its effectors. With this respect, mRNA studies from patients showed the coexistence of both wild-type and mutant transcripts in similar amounts. These observations might confirm that TNPO3 acts as a dimer when shuttling its cargoes to the nucleus and it is likely that the mutant protein fails in the formation of functional dimers. The authors also highlighted that the analysis of myomiRNAs, in particular miR-206, can be a promising biomarker for the clinical follow-up of LGMD D2 and for the interpretation of *TNPO3* uncertain gene variants (VUS).

The paper from Veneruso et al. from the child neurology group from Gaslini Children Hospital assessed the yield of myopathologic assessment in the diagnostic algorithm of neonatal hypotonia through a retrospective analysis of 197 infants. These findings revealed that 46% of biopsies showed unspecific alterations, while in 54% of cases, the biopsy proved to be decisive in orientating the genetic diagnosis, or in dressing up a diagnostic hypothesis. In particular, about 20% of biopsies revealed the picture of congenital muscular dystrophy or congenital myopathy, and the remaining 20% showed features of a metabolic myopathy mostly affecting oxidative metabolism. In the remaining 10%, the histopathological study indicated the presence of neurogenic damage. The group in which the muscle biopsy showed the highest degree of agreement with the genetic results was the congenital myopathies group: among 19 histological samples indicative of congenital myopathy, in 10 cases (53%), the data were confirmed by subsequent genetic testing, showing a high accuracy of the biopsy findings. The ultrastructural investigation with electron microscopy also proved fundamental in this group, particularly in biopsies from patients with age <6 months where the muscle fibers are small in size. In the other groups, the concordance rate between the biopsy and genetic findings was 42% for congenital muscular dystrophies, 38% for neurogenic myopathies, and 21% for metabolic myopathies. As a whole, the author highlight that normal biopsies are still to be taken into account in ruling out a primitive muscle condition and orientate the clinicians toward differential diagnosis.

The paper from Gaina et al. highlights that the combination of protein expression muscle studies and molecular analysis improves the mutational characterization of Duchenne and Becker muscular dystrophies. The aim of the Gaina study was to investigate the relationship between the type, size, and location of the mutation that occurs in the DMD gene and their effect on dystrophin protein expression in a cohort of 40 male patients with dystrophinopathy. They evaluated the expression of dystrophin by immunofluorescence and immunoblotting. The correlation between protein and genetic data allowed to compare the diagnoses predicted by protein analysis alone with that obtained by molecular analysis in order to check the applicability of the frameshift theory and to identify exceptions from the reading frame rule. Immunohistochemical findings and exon deletions were not in accordance only in two cases, resulting in a reading frame theory adherence of 95%. Such exceptions from the reading frame rule have been reported previously. This work shows that the DNA translational open reading frame and the location of the mutation both influence the expression of dystrophin and the disease severity phenotype. The authors also proposed an algorithm for the characterization of dystrophinopathy patients.

Mauri et al. evaluated a cohort of patients with Ryanodine receptor type 1-related congenital myopathy (RYR1-RCM), focusing on four patients who showed a severe congenital phenotype and underwent a comprehensive characterization at a few months of life including histopathology, electromyography (EMG), and muscle MRI. Indeed, only an advanced diagnostic strategy could lead to adequate support and provide possible therapeutic strategies at a very young age. In two out of the four patients, a muscle biopsy was performed in the first days of life and electron microscopy was carried out, detecting typical features of congenital RYR1 myopathy.

Lupica et al. explored the diagnostic challenges in Multiple Acyl-CoA Dehydrogenase Deficiency (MADD) through a retrospective study on patients with a diagnosis of vacuolar myopathy with lipid storage followed in their neuromuscular unit in the last 20 years. Multiple acyl-CoA dehydrogenase deficiency (MADD) is an autosomal recessive disorder of fatty acid oxidation due to deficiency of the mitochondrial electron transfer chain, with a favorable response to riboflavin supplementation. In all patients, a muscle biopsy was informative, showing invariably a vacuolar lipid storage myopathy, suggesting its crucial role in the diagnosis.

Laflamme et al. highlighted the role of muscle biopsy in the identification of a homozygous deep intronic mutation altering the splicing of nebulin gene (*NEB*). *NEB* mutations are known to be responsible for about 50% of nemaline myopathy cases. Nebulin is a giant protein that is formed integrally with the sarcomeric thin filament. This complex gene is under extensive alternative splicing giving rise to multiple isoforms. In this study, they report a 6-year-old boy presenting with general muscular weaknesses. Identification of rod-shaped structures in the patient biopsy raised doubt about the presence of nemaline myopathy. The authors suggested that the mutation in the intron 144 created a new splicing site strong enough to compete with the natural splicing site next to the mutation. The similar strength of the new and natural splicing sites led them to believe that both sites could be used equally. The relative diminution of nebulin isoforms in the biopsy compared with the normal control is likely to be attributed to the creation of an early stop codon generated by the frameshift, when the new splicing site is used. Most of the time, this situation is known to expose the mutant mRNA to degradation by non-sense-mediated decay. The alternative splicing mechanistic of the nebulin is very complex and not well-understood. This study suggests that although a good percentage of isoforms are still expressed in the patient, a decrease or disturbance in their ratios could have an impact on the proper function of this protein. Future discovery of other variants in *NEB*, and especially the ones that are in the alternatively spliced region will allow a better understanding of this complex giant protein. Indeed, finding variants affecting the alternative splicing of nebulin will make it possible to know more about the function of the different isoforms and allow us to clarify their respective implications in congenital nemaline myopathy.

Belkheir et al. provided the first report of a severe form of ßIV-spectrin deficiency with mitochondrial dysfunction. ßIV-spectrin is a protein of the spectrin family which is involved in the organization of the cytoskeleton structure. Mutations in the *SPTBN4* gene, coding for ßIV-spectrin, have been identified in seven individuals causing congenital myopathy, neuropathy, and central deafness. So far no mitochondrial dysfunction or cardiomyopathy has been reported in these patients. The authors described two individuals with a homozygous stop mutation in *SPTBN4* presenting with muscular hypotonia and atrophy, severe psychomotor developmental arrest, and cardiomyopathy. The biochemical examination disclosed the presence of a mitochondrial dysfunction probably linked to a destabilization of the cytoskeleton and thus to misclustering of ion channels. The resulting process could cause altered calcium homeostasis and increased calcium influx inside mitochondria inducing calpains activation. Furthermore, increased calcium influx leads to the opening of MPTP and thus swollen mitochondria and metabolic collapse of the latter. Complex I produces ROS which leads to increased calcium concentration and cell death. ROS are produced in complex I. Increasing oxidative stress leads to cell stress and to the liberation of calcium from the ER.

Finally, Cavalli et al. described the first Italian family with *MATR3*-Related Distal Myopathy, stressing the importance of muscle biopsy for the diagnosis and the characterization of this poorly understood disease. Mutations in *MATR3* are associated with distal myopathy with vocal cord and pharyngeal weakness (VCPDM), as well as familiar and sporadic motor neuron disease. To date, 12 VCPDM families have been described in the literature. VCPDM turns out to be a quite homogenous late-onset distal myopathy. With regard to myopathology, myopathic changes are largely predominant, being reported in 82% of biopsies. Rimmed vacuoles are observed in 59% of them. On the other hand, type grouping and other neurogenic changes are found in 14% of biopsies. Notably, type 2 fiber predominance found in the muscle biopsy of the patient reported by Cavalli et al. and commonly associated with neurogenic changes, might suggest a motor neuron involvement. MATR3-related pathology, encompassing myopathy and motor neuron disease, represents an illustrative example of multisystem proteinopathy (MSP).

In conclusion with this Research Topic, we reaffirm the diagnostic role of the muscle biopsy in different groups of inherited myopathies, and stress the importance of the research field of myopathology, where a multidisciplinary and complementary approach, including all different examinations, is often needed to give a definite diagnosis to patients. The myopathological study increases the understanding of the pathologic basis of several neuromuscular disorders, that are related to clinical outcomes and potentially disclose future therapeutic targets.

## Author contributions

All authors listed have made a substantial, direct, and intellectual contribution to the work and approved it for publication.

## Funding

GM was supported by FMM-Fondazione Malattie Miotoniche, Milan, Italy. CF was supported by AFM Telethon Trampoline Grant 22034.

## Conflict of interest

The authors declare that the research was conducted in the absence of any commercial or financial relationships that could be construed as a potential conflict of interest.

## Publisher's note

All claims expressed in this article are solely those of the authors and do not necessarily represent those of their affiliated organizations, or those of the publisher, the editors and the reviewers. Any product that may be evaluated in this article, or claim that may be made by its manufacturer, is not guaranteed or endorsed by the publisher.

